# A multicenter, clinically interpretable prediction model for malignancy risk in C-TIRADS 3–4 thyroid nodules

**DOI:** 10.3389/fonc.2026.1795852

**Published:** 2026-05-18

**Authors:** Wei Liu, Quan Xie, Chongmei Liu, Bolin Chen, Xilu Yang, Lingge Yang, Huizhi Yu

**Affiliations:** 1 Department of Pathology, Yueyang People’s Hospital of Hunan Normal University, Yueyang, China; 2 Graduate Collaborative Training Base of Yueyang, Hengyang Medical School, University of South China, Hengyang, China; 3 Graduate Collaborative Training Base of Hunan Cancer Hospital, Hengyang Medical School, University of South China, Hengyang, China; 4 The Second Department of Thoracic Oncology, The Affiliated Cancer Hospital of Xiangya School of Medicine, Central South University/Hunan Cancer Hospital, Changsha, China; 5 Department of Respiratory and Critical Care Medicine, Yueyang People’s Hospital of Hunan Normal University, Yueyang, China; 6 Department of Nutrition, Yueyang People’s Hospital of Hunan Normal University, Yueyang, China

**Keywords:** Clinical decision support, machine learning, nomogram, risk stratification, thyroid nodules, ultrasound

## Abstract

**Objectives:**

To develop and validate clinically interpretable prediction models that integrate ultrasonographic features and laboratory indicators for malignancy risk assessment in thyroid nodules, and to evaluate their potential value in supporting clinical decision-making.

**Methods:**

This was a multicenter retrospective study. 631 thyroid nodules from 478 patients at Yueyang People’s Hospital and 193 thyroid nodules from 144 patients at Hunan Cancer Hospital were included. Data from Yueyang People’s Hospital was used as the modeling cohort, randomly divided into a training set and an internal validation set (approximately 7:3) based on patient data. Data from Hunan Cancer Hospital served as an independent external validation set. Logistic regression, random forest, support vector machine(SVM), XGBoost, and LightGBM models were constructed based on ultrasound imaging features and clinical laboratory indicators, and their predictive performance was evaluated in the internal and external validation sets.

**Results:**

In the internal validation cohort, the logistic regression model achieved an area under the receiver operating characteristic curve (AUC) of 0.924 (95% CI: 0.881–0.959), comparable to the support vector machine (AUC = 0.922) and other machine learning models. In the external validation cohort, the AUCs ranged from 0.871 to 0.929, with logistic regression demonstrating strong and stable discriminative performance (AUC = 0.929, 95% CI: 0.887–0.965). Pairwise comparisons using the DeLong test showed that no statistically significant differences were observed between models in the internal validation cohort after Bonferroni correction. In contrast, in the external validation cohort, logistic regression demonstrated significantly higher AUCs than support vector machine, random forest, XGBoost, and LightGBM after correction. The logistic regression model also demonstrated balanced classification performance, good calibration, and favorable clinical utility in decision curve analysis. A nomogram was constructed based on this model for individualized malignancy risk assessment.

**Conclusions:**

This study demonstrates that, under strict variable selection, a logistic regression model based on routine clinical and ultrasound information can provide a stable and clinically interpretable risk assessment tool for C-TIRADS 3–4 thyroid nodules.

## 1 Introduction

Thyroid nodules are frequently encountered in routine endocrine care, and their detection has increased markedly with the broader use of high-resolution ultrasonography. While most nodules demonstrate benign or indolent behavior, the global number of newly diagnosed thyroid cancers has continued to rise, a trend that largely reflects intensified diagnostic activity rather than a parallel increase in disease-specific mortality (1–4). As a result, contemporary clinical management faces a fundamental challenge: not the identification of thyroid nodules, but the accurate stratification of malignancy risk to guide appropriate diagnostic and therapeutic interventions while avoiding unnecessary procedures (3, 5).

Several ultrasound-based classification systems, such as those from the American Thyroid Association, ACR TI-RADS, EU-TIRADS, and the Chinese Thyroid Imaging Reporting and Data System (C-TIRADS), have been developed to support malignancy risk assessment in thyroid nodules (5–9). While these systems offer structured frameworks, they primarily rely on predefined morphologic features and rule-based scoring schemes. These approaches have limitations in their ability to capture the complex interactions among clinical, laboratory, and imaging variables, leading to variability in performance across different populations and clinical settings (7, 8). In particular, C-TIRADS category 3–4 nodules represent an intermediate-risk “gray zone”, in which management decisions remain controversial and often heterogeneous in real-world practice.

The growing complexity of managing these intermediate-risk nodules emphasizes the urgent need for better tools to support clinicians in making informed, individualized decisions. Recently, data-driven modeling techniques have been introduced to integrate information from multiple sources, with the goal of improving malignancy risk evaluation in thyroid nodules (10, 11). However, many existing models depend heavily on high-dimensional imaging features, radiomics, or complex algorithms, which often lack transparency and interpretability, limiting their clinical applicability. In particular, such models often have insufficient external validation, making them difficult to apply in routine clinical practice (10, 12, 13).

There is, therefore, a pressing need for a clinically deployable, interpretable, and robust prediction model that focuses specifically on intermediate-risk thyroid nodules and is based on routinely available clinical and ultrasonographic data. In this multicenter retrospective study, we aimed to develop and externally validate prediction models that integrate conventional ultrasound features and laboratory indicators to assess malignancy risk in C-TIRADS 3–4 thyroid nodules. By systematically comparing several modeling approaches and emphasizing both interpretability and clinical utility, our goal was to identify a robust, easy-to-use risk estimation tool that complements existing ultrasound-based stratification systems and supports individualized management decisions.

## 2 Materials and methods

**Figure 1:**
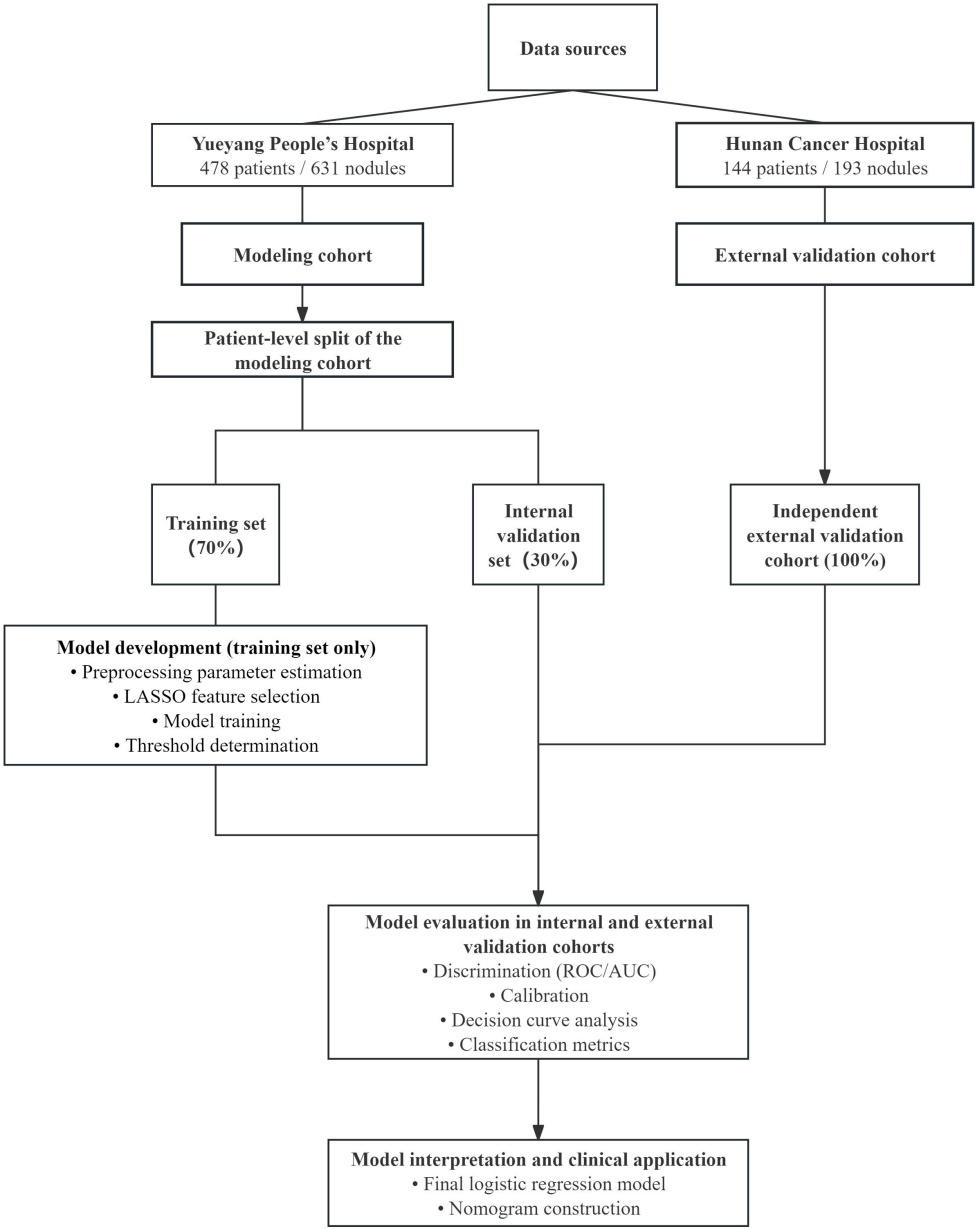
Study design and analytical workflow. Data from Yueyang People’s Hospital were used as the modeling cohort and were split at the patient level into training and internal validation sets at an approximate ratio of 7:3. Data from Hunan Cancer Hospital served as an independent external validation cohort. All model development procedures, including preprocessing parameter estimation, LASSO feature selection, model fitting, and threshold determination, were performed using the training set only. The trained models were evaluated in the internal and external validation cohorts using discrimination, calibration, decision curve analysis, and classification metrics. The final logistic regression model was further visualized as a nomogram for individualized malignancy risk assessment.

To facilitate an overall understanding of the study design and analytical workflow, a schematic overview of the study is presented in **Figure 1**.

### 2.1 Patients

Patients with thyroid nodules were retrospectively identified from Yueyang People’s Hospital and Hunan Cancer Hospital over the period from January 2024 to September 2025. Preoperative thyroid ultrasonography was performed in all cases, and the final benign or malignant status of each nodule was confirmed by postoperative histopathological examination. Following the application of predefined inclusion and exclusion criteria, the study population comprised 824 thyroid nodules from 622 patients, including 631 nodules from 478 patients treated at Yueyang People’s Hospital and 193 nodules from 144 patients treated at Hunan Cancer Hospital. The study protocol was reviewed and approved by the ethics committees of all participating institutions, and the requirement for written informed consent was waived in accordance with national regulations and institutional policies.

#### 2.1.1 Inclusion criteria

1. Patients underwent thyroid ultrasound examination, and the nodules were assessed as C-TIRADS category 3 or 4;
2. Patients had complete and evaluable ultrasound imaging data, with image quality meeting the analysis requirements;
3. Patients underwent surgical resection and obtained a clear postoperative pathological diagnosis;
4. Patients had available clinical and laboratory data of sufficient completeness for analysis, although limited missing values in some variables were permitted and subsequently handled during data preprocessing.

#### 2.1.2 Exclusion criteria

1. Patients who had previously received thyroid-related treatments, including surgery, radiotherapy, or other local therapies;
2. Patients lacking key clinical data or whose ultrasound imaging features could not be fully evaluated;
3. Cases with unclear pathological diagnoses or missing follow-up information.

### 2.2 Ultrasound imaging features and laboratory indicators

Patient-level clinical information and laboratory test results were extracted from electronic health records maintained at Yueyang People’s Hospital and Hunan Cancer Hospital. All data were routine preoperative tests and included in the statistical analysis. All patients underwent standardized thyroid ultrasound examinations.

Ultrasound images were acquired by sonographers at each participating center as part of routine clinical practice. For the purpose of this study, ultrasound image interpretation was independently performed by two associate chief physicians specialized in thyroid ultrasound imaging, each with extensive experience in thyroid nodule evaluation. The reviewers were blinded to pathological results and clinical outcomes during the image assessment process. All nodules were evaluated according to the Chinese Thyroid Imaging Reporting and Data System (C-TIRADS) framework. In cases of disagreement between the two reviewers, the final interpretation was determined by adjudication from a third senior associate chief physician who was not involved in the initial image evaluation.

Ultrasound imaging features were evaluated according to C-TIRADS (14). Analytical features included nodule echogenicity, morphology, margins, aspect ratio, calcification, multifocality, and echogenic foci. Nodule size was recorded as the largest diameter observed on ultrasonographic examination.

### 2.3 Data preprocessing

Continuous variables were first truncated at the 1st and 99th percentiles to reduce the impact of extreme values, and then standardized using the Z-score method. Incomplete records were managed using a chained-equations multiple imputation strategy (MICE). The assumption of linearity between continuous predictors and the logit of the outcome was examined using restricted cubic spline (RCS) analyzes for selected variables, including age, nodule size, and thyroid-stimulating hormone (TSH) (15). As no meaningful departure from linearity was detected within clinically relevant ranges, these variables were subsequently modeled as linear terms. Although patients were required to have sufficiently complete data for inclusion, a small proportion of missing values remained in certain variables, which were handled using multiple imputation by chained equations (MICE).

### 2.4 Model development

Given that this was a retrospective multicenter study based on consecutively available clinical cases, no prospective sample size target was prespecified before data collection. Nevertheless, the adequacy of the available sample for prediction model development was evaluated retrospectively in light of contemporary methodological recommendations. A total of 824 nodules were included, of which 422 were malignant events. With 9 predictors retained in the final model, the events-per-variable ratio was approximately 46.9. Although EPV alone is not sufficient to establish sample size adequacy, this relatively large number of events in relation to model complexity supports a lower risk of severe overfitting and suggests that the dataset was reasonably adequate for stable model development. In addition, model robustness and generalizability were further evaluated through internal validation, external validation, and bootstrap resampling, providing complementary evidence for the stability of the developed prediction models (16).

To minimize potential clustering effects arising from patients with multiple nodules, dataset partitioning was performed at the patient level rather than the nodule level, ensuring that nodules from the same patient were not distributed across different datasets and thereby preventing information leakage. All preprocessing steps were implemented using information from the training cohort, and the corresponding transformation parameters were subsequently applied to the internal and external validation cohorts to avoid information leakage. In addition, a sensitivity analysis restricted to patients with a single thyroid nodule was conducted to further evaluate the potential impact of intra-patient correlation on model performance.

Using data from the modeling cohort, we developed and assessed several prediction models to estimate malignancy risk. Clinical data, ultrasound features, and laboratory indicators were collected as candidate predictor variables. Univariate analyzes were performed solely for descriptive purposes to characterize clinical differences between benign and malignant thyroid nodules.

A total of 20 candidate variables, including clinical characteristics, ultrasonographic features, and laboratory indicators, were initially considered for model development. All predefined candidate variables were simultaneously entered into a logistic regression–based least absolute shrinkage and selection operator (LASSO) model for feature selection. The penalty parameter (λ) was determined using 10-fold cross-validation, and the λ1se criterion was adopted to obtain a more parsimonious and stable model. Variables with non-zero coefficients at λ1se were retained for subsequent multivariable analysis. The detailed variable retention process across different stages of model development is provided in **Supplementary Table 1**.

To assess potential multicollinearity among predictors, Spearman correlation coefficients and corresponding p-values were calculated in the training cohort and visualized using a heatmap. In addition, variance inflation factors (VIFs) were computed based on the final multivariable logistic regression model.

Five predictive modeling approaches were developed, including logistic regression, support vector machine (SVM), random forest (RF), XGBoost, and LightGBM. For all models, hyperparameter tuning was performed exclusively within the training cohort using a grid search strategy combined with 10-fold cross-validation. The optimal hyperparameter combination for each model was selected by maximizing the mean area under the receiver operating characteristic curve (AUC) across cross-validation folds.

The hyperparameter search space was predefined based on prior literature and practical considerations. Specifically, for logistic regression, the regularization strength (C), penalty type, and solver were explored. For the random forest model, the number of trees, maximum tree depth, minimum samples required for node splitting, and minimum samples per leaf were tuned. For the SVM model with a radial basis function kernel, the cost parameter (C) and kernel coefficient (gamma) were optimized. For XGBoost, the number of estimators, learning rate, maximum tree depth, subsample ratio, and colsample_bytree were tuned. For LightGBM, the number of estimators, learning rate, number of leaves, maximum tree depth, and subsample ratio were optimized. Detailed hyperparameter search ranges and the final selected parameters for each model are provided in **Supplementary Table 2** (14, 17, 18).

After selection of the optimal hyperparameters, each model was refitted on the full training dataset and subsequently evaluated in the internal and external validation cohorts, which were strictly held out from all stages of model development, to provide unbiased estimates of predictive performance (17).

### 2.5 Model evaluation and clinical application

All cross-validation procedures were conducted within the training cohort only and were used to support model development, including LASSO penalty selection and robustness assessment. The internal validation cohort and the external validation cohort were reserved exclusively for independent model evaluation.

Discrimination was primarily quantified by the area under the receiver operating characteristic curve (AUC), with accuracy, sensitivity, specificity, precision, and F1 score reported as complementary metrics (19, 20).

Pairwise comparisons of AUCs between models were performed using the DeLong test. To account for multiple comparisons, Bonferroni correction was applied, and a corrected significance threshold of P < 0.005 was considered statistically significant.

Clinical applicability was explored using decision curve analysis (DCA) across a range of risk thresholds. Calibration performance was assessed using calibration curves and Brier scores, and bootstrap resampling was performed for internal validation to estimate optimism-adjusted performance and evaluate model stability. For the model selected as the final model after comparative evaluation, a nomogram was subsequently constructed for individualized risk estimation.

A nomogram was developed based on the final logistic regression model, translating regression coefficients into a visual scoring scheme for individualized estimation of thyroid nodule malignancy risk.

Data analysis was conducted using R (version 4.5.1; R Foundation for Statistical Computing, Vienna, Austria) and Python (version 3.x; Python Software Foundation, Wilmington, DE, USA). A two-sided P value < 0.05 was considered statistically significant unless otherwise specified.

## 3 Results

### 3.1 Baseline characteristics

The study cohort consisted of 622 patients with 824 thyroid nodules, including 422 malignant nodules (51.2%) and 402 benign nodules (48.8%). The observed proportion of malignant nodules was consistent with that reported in prior investigations of C-TIRADS 3–4 nodules.

Among the enrolled nodules, 317 were classified as C-TIRADS 3, of which 27 were malignant (8.5%). In comparison, nodules classified as C-TIRADS 4 showed substantially higher malignancy rates, including 58.0% for C-TIRADS 4a, 86.0% for C-TIRADS 4b, and 94.6% for C-TIRADS 4c, demonstrating a progressive increase in malignancy risk across higher C-TIRADS categories. The distribution of malignancy across C-TIRADS categories is summarized in **Supplementary Table 3**.

Patients were randomly allocated at the patient level to a training cohort and an internal validation cohort with an approximate 7:3 split. Cases from Hunan Cancer Hospital were reserved as an independent external validation cohort. Baseline demographic data, ultrasonographic characteristics, and laboratory measurements for the three cohorts are presented in **Supplementary Table 4**. Variations were noted in age, thyroid-stimulating hormone (TSH), and total thyroxine (TT4) across cohorts, while sex distribution and most ultrasonographic features showed overall similarity. All P values are provided for descriptive purposes.

### 3.2 Univariate analysis and LASSO-based variable selection

In the training cohort, univariate comparisons were performed to describe the distribution of clinical, ultrasonographic, and laboratory variables between benign and malignant nodules. Several ultrasonographic features and selected laboratory indicators showed statistically significant differences or trends toward significance (P < 0.10).

**Figure 2:**
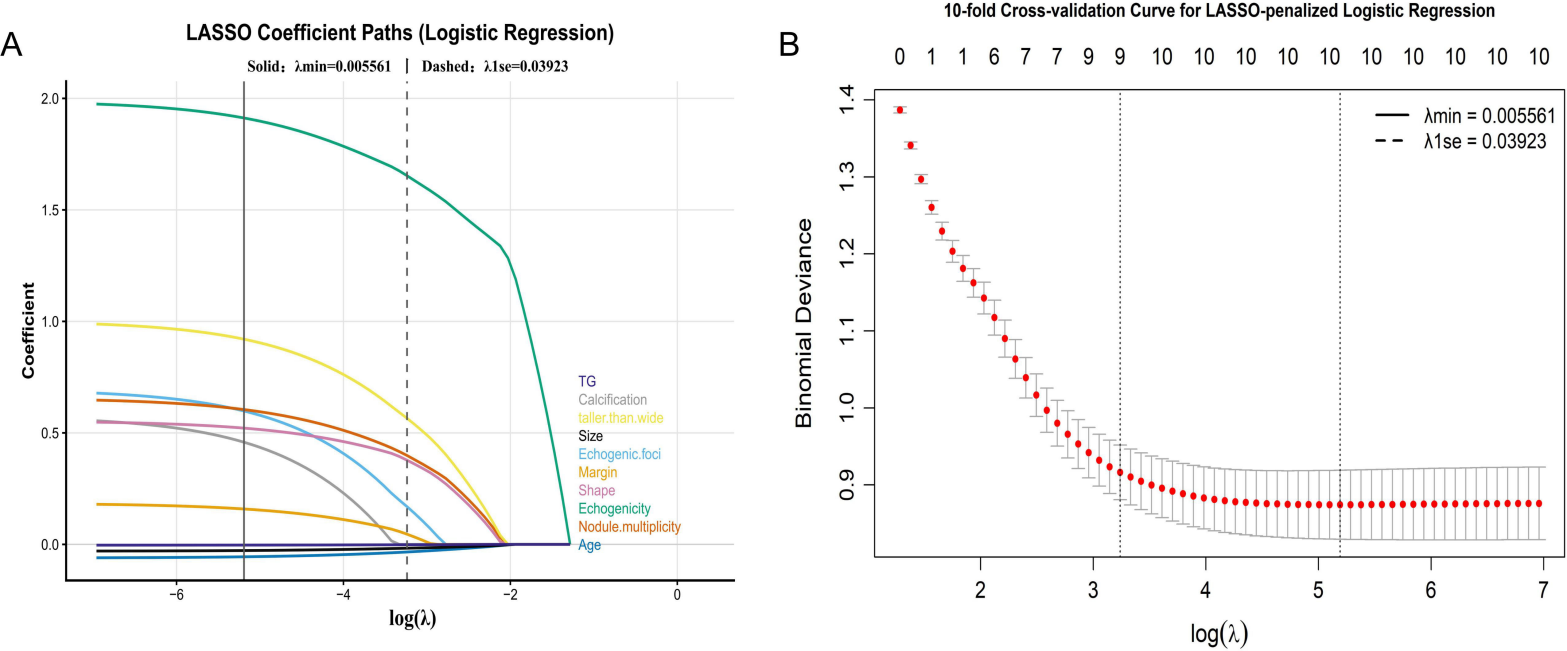
LASSO-based feature selection in logistic regression. **(A)** LASSO coefficient profiles of candidate predictors as a function of log(λ). **(B)** Selection of the optimal penalty parameter (λ) using 10-fold cross-validation based on binomial deviance. The vertical lines indicate λmin and λ1se, respectively.

All predefined candidate variables were entered into a logistic regression–based LASSO framework for feature selection. The penalty parameter (λ) was optimized through 10-fold cross-validation, resulting in the retention of nine predictors with stable non-zero coefficients at λ_1_se (**Figures 2A, B**). These variables were subsequently adopted as the common input set for all predictive models. Correlation analysis showed that most pairwise associations among the selected predictors were weak to moderate, with no strong correlations observed. Consistently, VIF analysis indicated that all predictors had low values (range, 1.062–1.230), suggesting that multicollinearity was unlikely to affect model construction. The correlation heatmap and VIF results are presented in **Supplementary Figure 4** and **Supplementary Table 5**, respectively.

**Figure 3:**
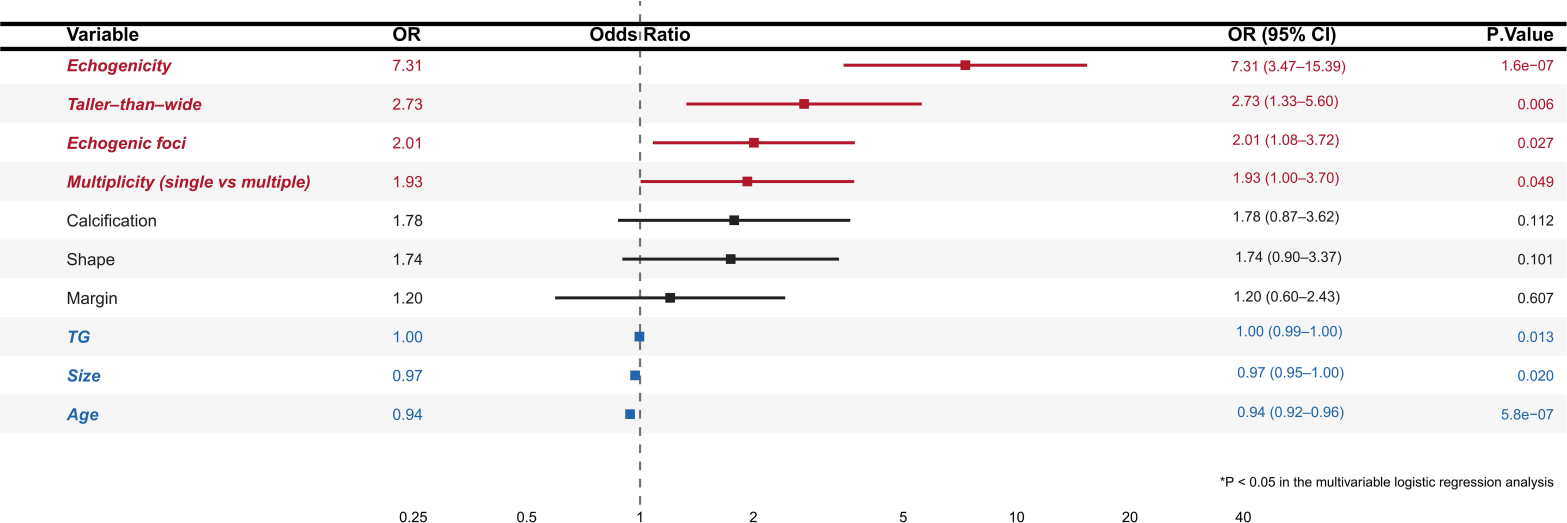
Forest plot of multivariable logistic regression. The forest plot illustrates the association between candidate predictors and the risk of malignancy in thyroid nodules based on a multivariable logistic regression model. Point estimates represent odds ratios (ORs), and horizontal lines indicate the corresponding 95% confidence intervals (95% CIs). The dashed vertical line denotes the null reference (OR = 1). ORs are presented per one-unit increase in each predictor variable based on their original measurement scales. For categorical variables, ORs are interpreted relative to the reference category. OR > 1 indicates an increased risk of malignancy, whereas OR < 1 indicates a decreased risk. The right panel reports the OR (95% CI) and corresponding p-value for each variable.

A multivariable logistic regression model was fitted using the selected predictors. The relationships between each variable and malignancy risk are summarized as odds ratios with corresponding 95% confidence intervals in the forest plot (**Figure 3**).

### 3.3 Discriminative performance of prediction models

**Figure 4:**
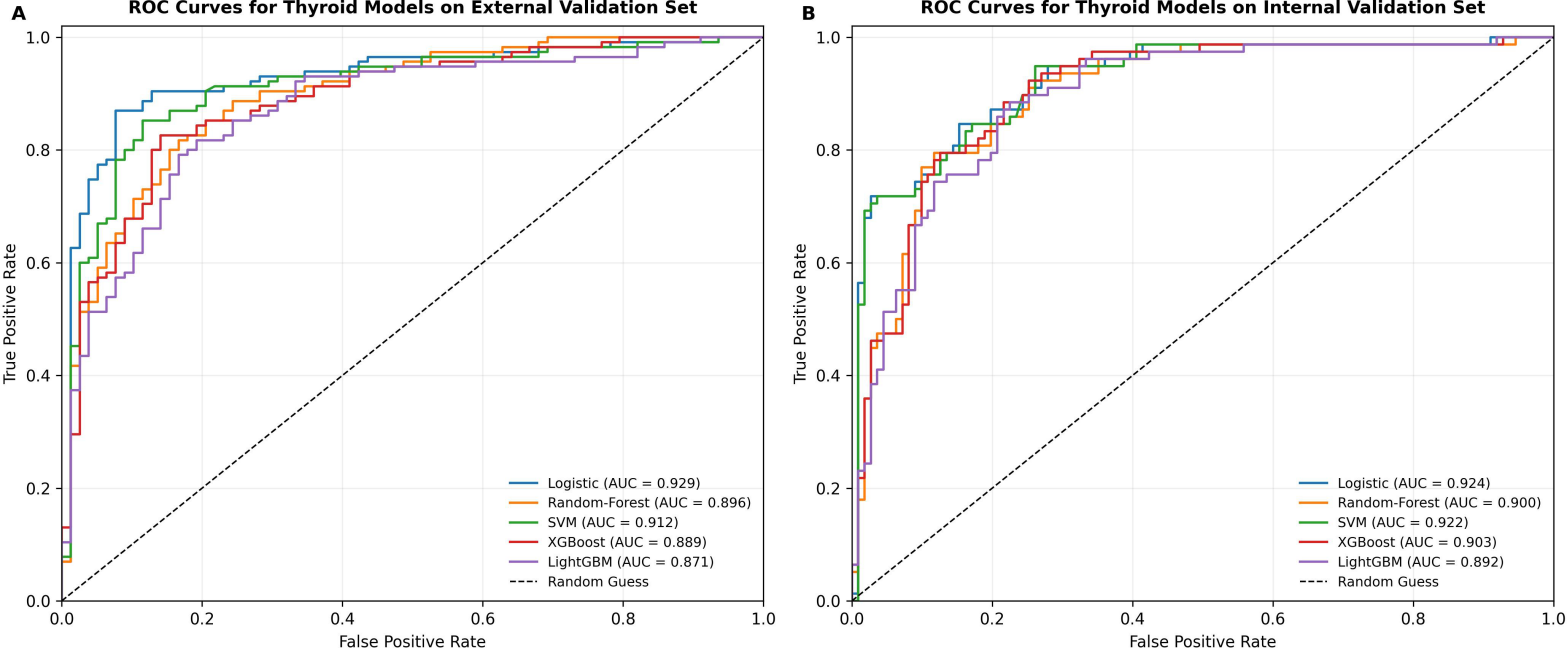
ROC curves of predictive models on the validation set. **(A)** Receiver operating characteristic (ROC) curves of logistic regression, random forest, SVM, XGBoost, and LightGBM models evaluated in the external validation cohort. **(B)** ROC curves for the same five models assessed in the internal validation cohort. The x-axis denotes the false positive rate, and the y-axis denotes the true positive rate. The diagonal dashed line indicates performance equivalent to random classification. AUC values for each model are provided in the legend.

Using the training cohort, five predictive models were developed, including logistic regression, SVM, random forest, XGBoost, and LightGBM. After hyperparameter optimization within the training cohort and refitting on the full training set, model performance was evaluated in the internal and external validation cohorts. Receiver operating characteristic (ROC) curves are presented in **Figure 4**, with corresponding performance measures summarized in **Table 1**.

**Table 1:** Performance of the final tuned models in the internal and external validation cohorts.

Model	External AUC (95% CI)	External accuracy	External sensitivity	External specificity	External precision	External F1-score
Logistic	0.929 (0.887–0.965)	0.850	0.913	0.756	0.847	0.879
SVM	0.912 (0.865–0.952)	0.860	0.913	0.782	0.861	0.886
Random Forest	0.896 (0.847–0.939)	0.834	0.887	0.756	0.843	0.864
XGBoost	0.889 (0.838–0.933)	0.808	0.861	0.731	0.825	0.843
LightGBM	0.871 (0.817–0.921)	0.798	0.817	0.769	0.839	0.828
Model	Internal AUC (95% CI)	Internal accuracy	Internal sensitivity	Internal specificity	Internal precision	Internal F1-score
Logistic	0.924 (0.881–0.959)	0.825	0.872	0.793	0.747	0.805
SVM	0.922 (0.878–0.957)	0.815	0.897	0.757	0.722	0.800
Random Forest	0.900 (0.850–0.942)	0.804	0.885	0.748	0.711	0.789
XGBoost	0.903 (0.854–0.944)	0.815	0.833	0.802	0.747	0.788
LightGBM	0.892 (0.842–0.936)	0.804	0.821	0.793	0.736	0.776

Model performance was evaluated using six predefined metrics. All reported performance estimates were obtained from the independent internal and external validation cohorts. Cross-validation was conducted within the training cohort solely to support model development and was not used for reporting model performance.

In the internal validation cohort, AUC was 0.924 (95% CI, 0.881–0.959) for logistic regression, 0.922 (95% CI, 0.878–0.957) for SVM, 0.900 (95% CI, 0.850–0.942) for random forest, 0.903 (95% CI, 0.854–0.944) for XGBoost, and 0.892 (95% CI, 0.842–0.936) for LightGBM.

In the external validation cohort, the AUCs were 0.929 (95% CI, 0.887–0.965) for logistic regression, 0.912 (95% CI, 0.865–0.952) for SVM, 0.896 (95% CI, 0.847–0.939) for random forest, 0.889 (95% CI, 0.838–0.933) for XGBoost, and 0.871 (95% CI, 0.817–0.921) for LightGBM.

Pairwise comparisons of AUCs using the DeLong test showed that, after Bonferroni correction, no statistically significant differences were observed among the models in the internal validation cohort. In the external validation cohort, the logistic regression model achieved significantly higher AUCs than all other models, including support vector machine, random forest, XGBoost, and LightGBM (all Bonferroni-adjusted P < 0.005). Among the remaining models, the support vector machine showed a significantly higher AUC than LightGBM, while no statistically significant differences were observed between support vector machine and random forest or XGBoost after correction.

Because logistic regression demonstrated stable discrimination performance and provided an interpretable probability-based prediction model suitable for clinical application, further classification analysis was performed for this model. The classification threshold for the logistic regression model was determined based on ROC analysis in the training cohort. Specifically, the optimal probability threshold was identified using the maximum Youden index. According to this criterion, the optimal cut-off value was 0.490, which was subsequently applied to the internal and external validation cohorts for classification analysis (**Supplementary Figure 1**).

**Figure 5:**
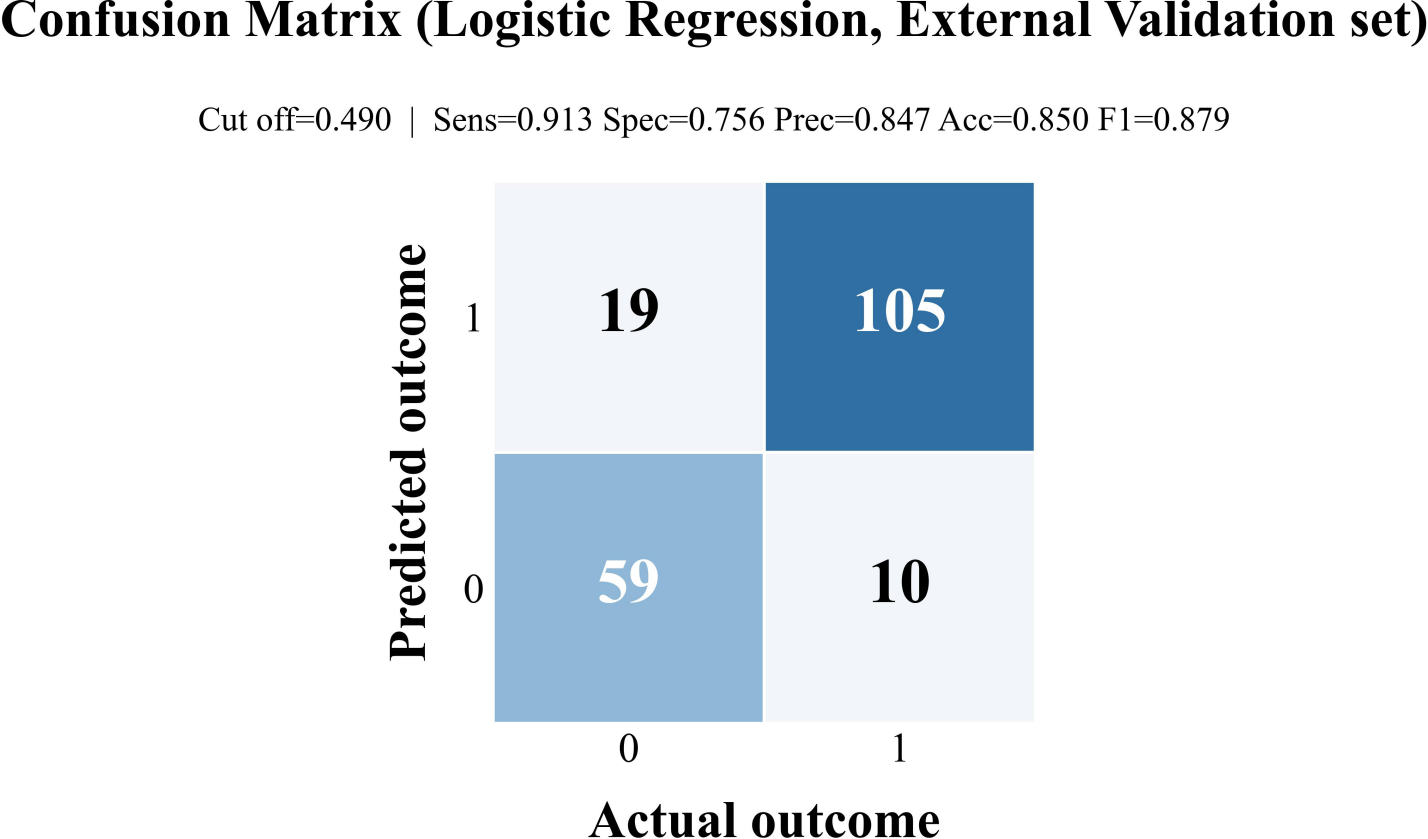
Confusion matrix for logistic regression in the external validation cohort. The confusion matrix summarizes the classification outcomes of the logistic regression model in the external validation cohort. Observed class labels are displayed along the horizontal axis, whereas predicted labels are shown on the vertical axis. Each cell reports the number of true positives (TP), true negatives (TN), false positives (FP), and false negatives (FN). The image additionally indicates the classification cutoff applied in this cohort, together with the corresponding sensitivity, specificity, accuracy, precision, and F1 score, providing an overview of model performance.

Confusion matrices for the logistic regression model in the external validation cohort are presented in **Figure 5**.

### 3.4 Decision curve analysis

**Figure 6:**
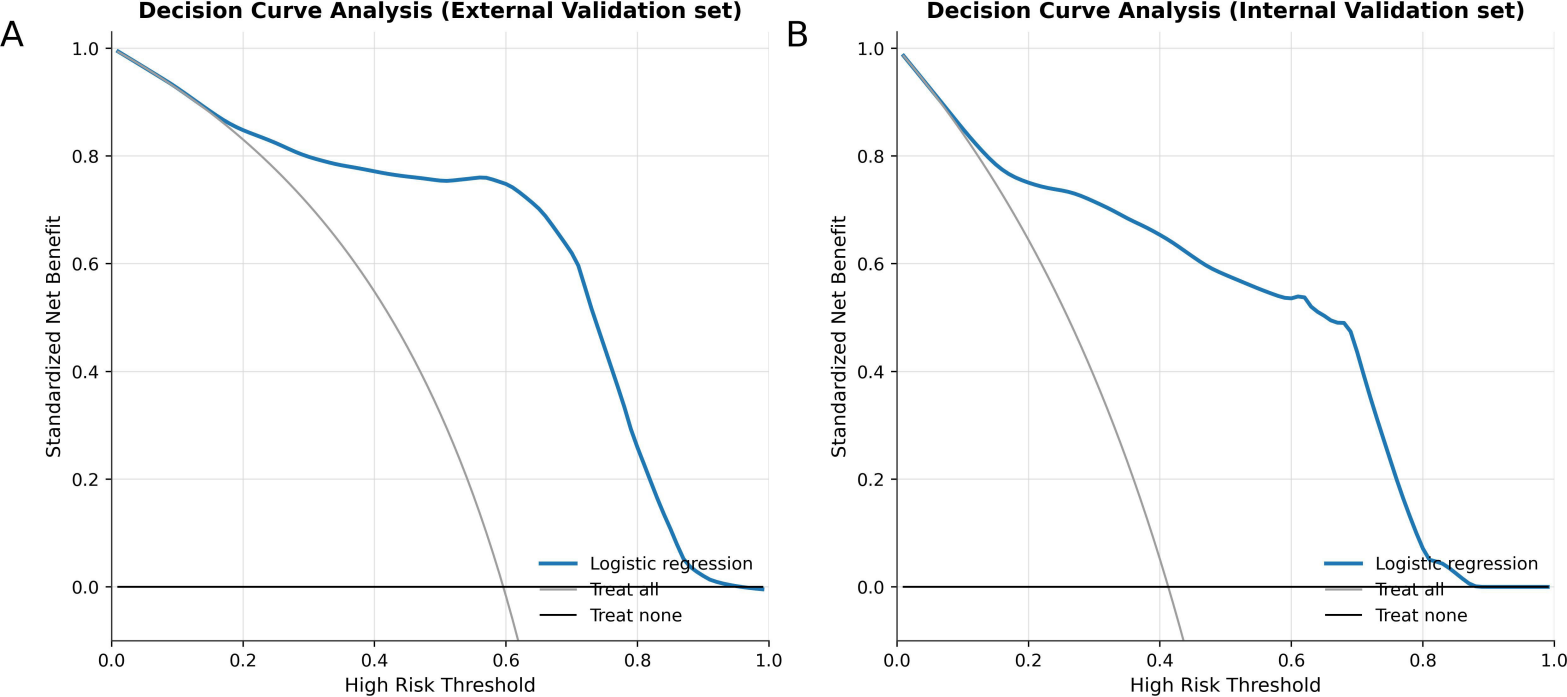
Decision curve analysis in the validation set. **(A)** Decision curve for the logistic regression model in the external validation cohort. **(B)** Decision curve for the logistic regression model in the internal validation cohort. Threshold probability is displayed on the x-axis, and standardized net benefit on the y-axis. The blue solid curve corresponds to the net benefit of the logistic regression model across threshold probabilities, while the gray and black reference lines denote the treat-all and treat-none strategies, respectively. Decision curve analysis illustrates the potential clinical value of applying the model at different decision thresholds in comparison with these reference strategies.

DCA was employed to evaluate model-based net benefit across clinically relevant threshold probabilities. Across both validation cohorts, the logistic regression model showed superior net benefit relative to the treat-all and treat-none reference lines, as depicted in **Figure 6**.

### 3.5 Calibration and validation of the logistic regression model

**Figure 7:**
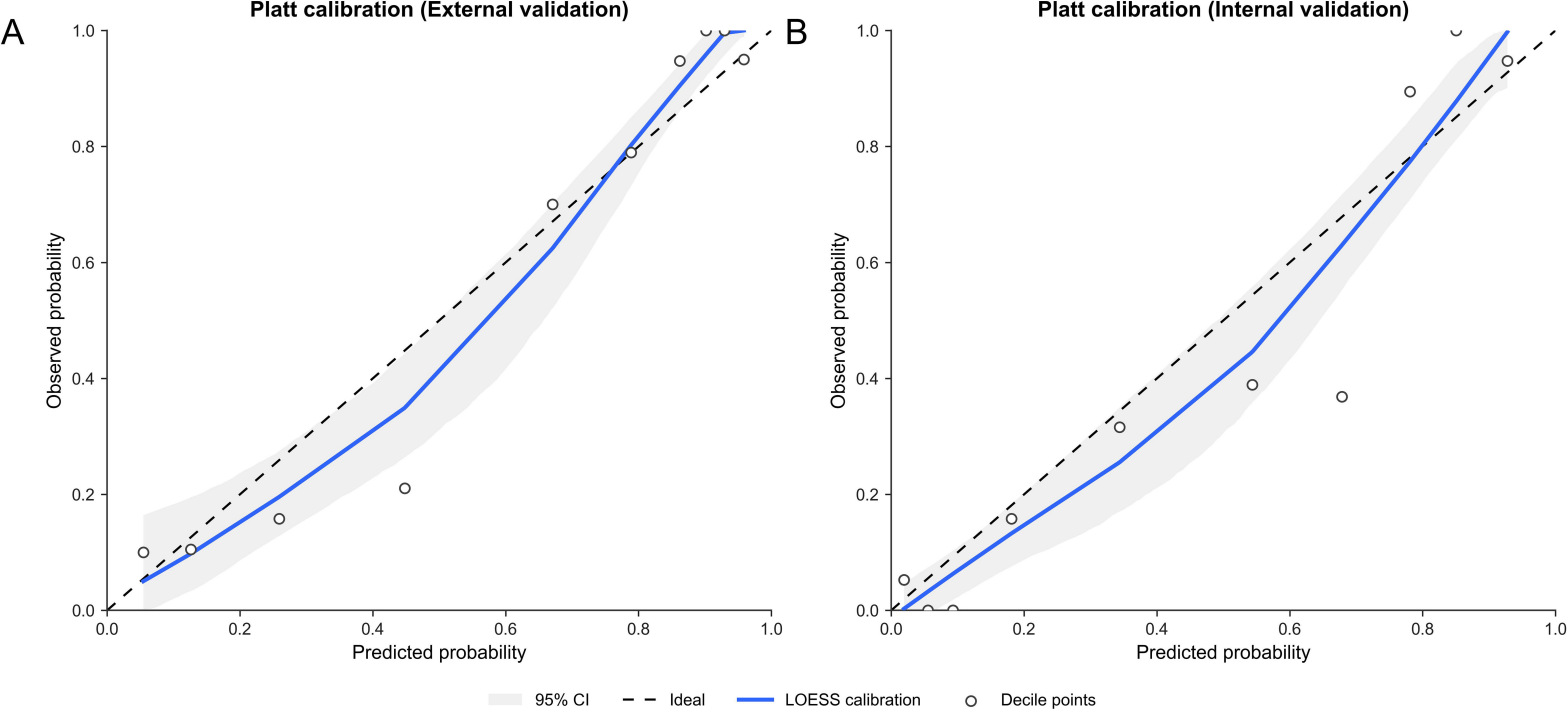
Platt-calibrated calibration plots in the validation cohorts. **(A)** External validation cohort. **(B)** Internal validation cohort. Calibration curves were generated after probability calibration using Platt scaling (sigmoid transformation) applied to the model outputs. The horizontal axis represents the predicted probability of malignancy, and the vertical axis represents the observed probability. The dashed black line indicates perfect calibration (ideal agreement between predicted and observed probabilities). The solid blue line represents the LOESS-smoothed calibration curve, illustrating the relationship between predicted and observed probabilities. The gray shaded area denotes the 95% confidence interval of the calibration curve, and the circles represent observed event rates within deciles of predicted risk. These plots assess the agreement between predicted probabilities and observed outcomes after model calibration.

Calibration of the logistic regression model was assessed using calibration curves and bootstrap resampling. The cross-validated AUC was 0.873, with a Brier score of 0.141. After bootstrap correction, the AUC was 0.859. Calibration plots for the internal and external validation cohorts are shown in **Figure 7**, demonstrating agreement between predicted probabilities and observed outcomes.

### 3.6 Nomogram construction and validation

**Figure 8:**
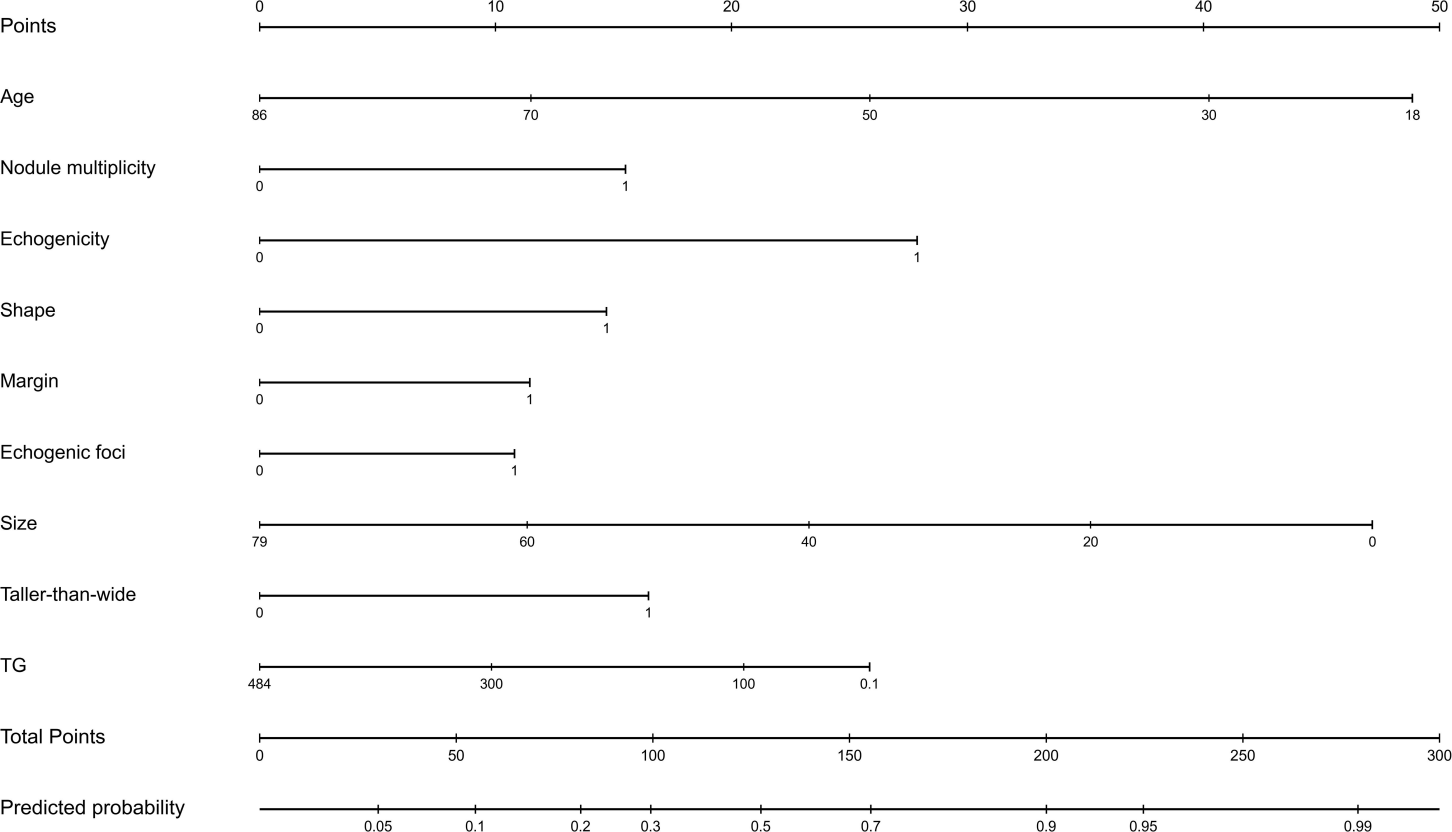
Nomogram for individualized estimation of malignancy risk in thyroid nodules. This nomogram, constructed based on a multivariate logistic regression model, is used to individually predict the malignancy risk of thyroid nodules. Each predictor variable (including age, multifocality, echogenicity, morphology, borders, punctate hyperechoic areas, nodule size, aspect ratio, and TG level) is assigned a point on its corresponding axis. The points are summed to obtain the total points. Based on the total point, a linear predictor and a predicted probability of malignancy are plotted on the lower axis, thus enabling a quantitative assessment of the malignancy risk for an individual patient.

A nomogram was derived from the final multivariable logistic regression model incorporating the nine selected predictors (**Figure 8**). Predictor-specific point assignments were summed to yield an individualized estimate of malignancy probability. Calibration performance of the nomogram was examined through bootstrap resampling. The analysis suggested acceptable calibration, comparable to that observed for the underlying logistic regression model.

## 4 Discussion

In this multicenter real-world study, we developed and systematically evaluated several prediction models for malignancy risk assessment in C-TIRADS 3–4 thyroid nodules, a subgroup characterized by substantial clinical uncertainty. Our results demonstrate that, after rigorous feature selection and integration of routinely available ultrasonographic and laboratory variables, a logistic regression–based model achieved stable discrimination, good calibration, and consistent clinical net benefit across both internal and external validation cohorts. The corresponding nomogram translates multivariable risk information into individualized probability estimates, providing a practical and interpretable tool to support clinical decision-making in intermediate-risk nodules.

From a methodological perspective, these findings underscore an important principle in clinical prediction modeling that is often underappreciated: when candidate predictors are limited in number, biologically meaningful, and derived from standardized clinical workflows, model performance is frequently constrained by information content rather than algorithmic complexity (21–23). In such structured clinical datasets, increasing model complexity does not necessarily lead to improved generalizability and may even compromise robustness when applied to external populations (24, 25). In contrast, regression-based models with transparent assumptions and parameter structures are more likely to maintain stable performance across centers. Importantly, after systematic hyperparameter tuning, logistic regression demonstrated superior performance in the external validation cohort, whereas no statistically significant differences were observed among models in the internal validation cohort. These findings indicate that, in structured clinical datasets with a limited number of predictors, increased model complexity does not necessarily translate into improved generalizability, and simpler models may provide competitive or even superior performance. Our findings are consistent with prior systematic evaluations of clinical prediction models and highlight the importance of aligning modeling strategies with data structure and intended clinical use, rather than prioritizing algorithmic sophistication alone (26, 27).

It is also important to recognize that C-TIRADS 3–4 nodules represent a clinical decision-making gray zone rather than a purely diagnostic problem (28–30). In this context, the primary objective is not to maximize classification accuracy but to achieve balanced risk estimation that supports individualized management decisions, including the avoidance of unnecessary fine-needle aspiration or surgical intervention while minimizing the risk of missing clinically significant malignancies (3, 5). Prediction models that provide well-calibrated probability estimates and transparent risk attribution may therefore be more clinically valuable than highly optimized classifiers focused solely on discrimination. The nomogram developed in this study was designed with this principle in mind and is intended to complement, rather than replace, existing ultrasound-based risk stratification systems and clinical judgment. In the present study, the selected probability threshold corresponded to the maximum Youden index, providing an optimal balance between sensitivity and specificity for malignancy risk stratification. In clinical practice, different probability thresholds may be considered depending on the clinical context. Lower thresholds may be preferred in scenarios where minimizing missed malignancies is prioritized, whereas higher thresholds may be considered when the goal is to reduce unnecessary interventions.

A further point that warrants clarification is the clinical context of the surgically treated C-TIRADS 3 nodules included in this study. These nodules should not be interpreted as representing the general surveillance population of C-TIRADS 3 nodules in routine practice. Rather, they constitute a selected subgroup of patients who entered the surgical pathway in real-world clinical care. Notably, the malignancy rate observed in C-TIRADS 3 nodules in this surgically treated cohort was 8.5%, which is higher than that typically reported in population-based surveillance cohorts, reflecting the surgically enriched nature of the study population. In clinical practice, surgical management of some C-TIRADS 3 nodules may be influenced by factors beyond the ultrasound category itself, such as interval growth during follow-up, compressive symptoms, coexistence of other suspicious nodules, patient preference, or broader clinical judgment. Accordingly, the prediction model developed in this study is primarily intended for preoperative malignancy risk stratification and management support in patients with C-TIRADS 3–4 nodules who have already undergone surgical selection or are being considered for invasive diagnostic or therapeutic intervention, rather than as a population-level screening tool or as a standalone basis for recommending surgery in routine follow-up patients with C-TIRADS 3 nodules.

At the variable level, serum thyroglobulin (Tg) was retained in the final model and showed an association with malignancy risk. The clinical interpretation of Tg is complex and influenced by multiple factors, including the reduction of functional thyroid tissue and potential assay interference from thyroglobulin antibodies (TgAb) (31, 32). Consequently, Tg should not be interpreted as a standalone diagnostic marker. From a modeling perspective, however, the inclusion of Tg illustrates how variables with limited individual diagnostic utility may still contribute complementary information when interpreted jointly with ultrasonographic and clinical features (31, 32). This observation further supports the rationale for multivariable, probability-based risk assessment rather than reliance on single features or rule-based scoring systems.

Importantly, the emphasis on interpretability in this study reflects practical considerations in real-world clinical settings. While complex machine learning models may achieve marginal performance gains, their limited transparency can hinder clinical adoption (33–35). In contrast, regression-based models with explicit variable contributions allow clinicians to better understand and trust model outputs, which is essential for integration into routine decision-making workflows (36, 37).

This study has certain limitations that warrant consideration. First, as a retrospective study, the analysis relied on routinely collected clinical data, which may introduce potential selection bias and information bias. In particular, the inclusion of surgically treated nodules with histopathological confirmation may have led to an overrepresentation of higher-risk lesions (38–40). As a result, the model is primarily applicable to diagnostic evaluation and management decision support rather than population-level screening. Second, ultrasonographic features were derived from routine clinical assessments; although standardized criteria and blinded review were applied, inter-operator variability cannot be completely eliminated (41, 42). Third, molecular biomarkers and high-dimensional quantitative imaging features were not incorporated, which may limit the characterization of biological heterogeneity (43–46). In addition, because multiple nodules could originate from the same patient, the dataset has a clustered structure that may potentially influence statistical estimation. To reduce information leakage, dataset splitting was performed at the patient level. Furthermore, we conducted a supplementary sensitivity analysis restricted to patients with a single thyroid nodule, and the predictive models demonstrated comparable discriminative performance in both the internal and external validation cohorts (**Supplementary Figure 2**), suggesting that the potential impact of clustering on model performance was limited. However, although external validation was performed, the sample size of the external cohort was relatively limited, which may affect the precision and stability of the performance estimates. Moreover, representative ultrasound images were provided to offer visual context for the C-TIRADS classification system (**Supplementary Figure 3**). However, not all illustrated imaging features were incorporated as predictors in the final model, as feature selection was guided by statistical significance and model stability considerations. Therefore, the included variables do not fully capture the entire spectrum of ultrasound characteristics used in routine clinical assessment, and the model should be interpreted as a complementary tool rather than a replacement for comprehensive ultrasound evaluation. Further validation through prospective, multicenter investigations is needed, together with exploration of multimodal data integration while maintaining model interpretability.

In summary, this study presents an interpretable and externally validated prediction model based on routinely available clinical and ultrasonographic information, providing a feasible approach for malignancy risk estimation in C-TIRADS 3–4 thyroid nodules. By emphasizing robustness, calibration, and clinical applicability, this model offers a pragmatic complement to existing risk stratification systems and may help support individualized management decisions while reducing unnecessary interventions.

## 5 Conclusions

This study presents a clinically interpretable and externally validated prediction model based on routinely available ultrasonographic and laboratory information for malignancy risk estimation in C-TIRADS 3–4 thyroid nodules. By emphasizing robustness, calibration, and clinical net benefit rather than algorithmic complexity, the proposed logistic regression–based nomogram offers a practical complement to existing ultrasound-based risk stratification systems. In particular, this tool may be most valuable in supporting individualized management decisions within the intermediate-risk setting, where diagnostic uncertainty is common and unnecessary invasive procedures remain a clinical concern.

## Data Availability

The datasets analyzed during the current study are not publicly available due to ethical restrictions, patient privacy considerations, and institutional data-protection requirements. Summary information derived from the datasets is provided in the main text and Supplementary Materials. Further inquiries regarding the datasets may be directed to the corresponding author. Requests to access the datasets should be directed to 18973093945@163.com and will be subject to approval by the relevant participating institutions.
