# Supplementary material for: A multicenter, clinically interpretable prediction model for malignancy risk in C-TIRADS 3–4 thyroid nodules
Source: Front Oncol. 2026 May 18;16:1795852. doi: 10.3389/fonc.2026.1795852 (PMC13222797; doi:10.3389/fonc.2026.1795852)
Supplement: Supplementary file 1 [file DataSheet1.pdf]

Supplementary Material

1 Supplementary Figures and Tables

1.1 Supplementary Figures

Supplementary Figure S1. Determination of the optimal probability threshold using the Youden index in the training cohort.

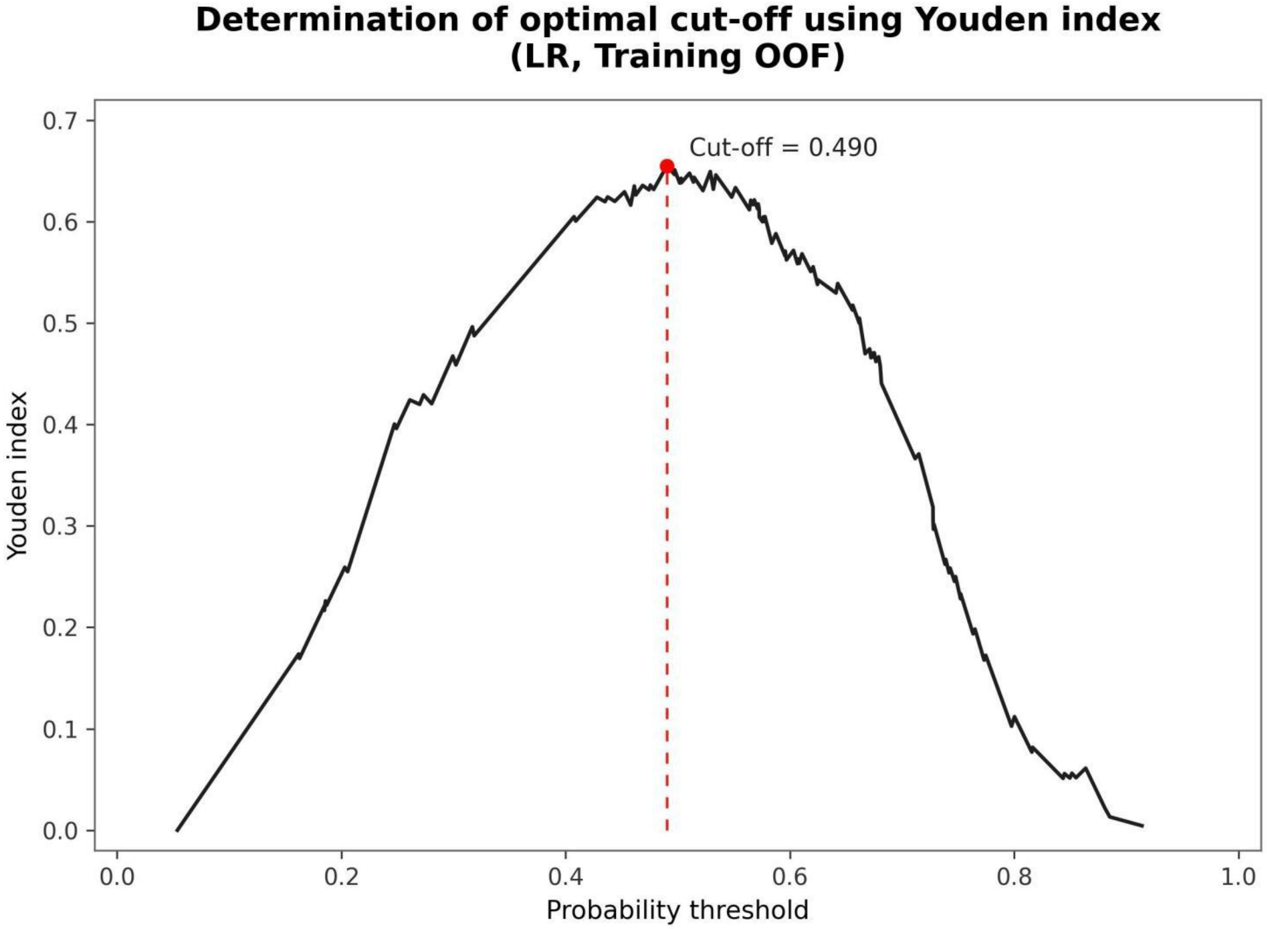

Supplementary Figure S1. The relationship between the probability threshold and the corresponding Youden index was evaluated. The vertical dashed line indicates the selected probability threshold (0.490), corresponding to the maximum Youden index.

Supplementary Figure S2. ROC curves in the single-nodule subgroup.

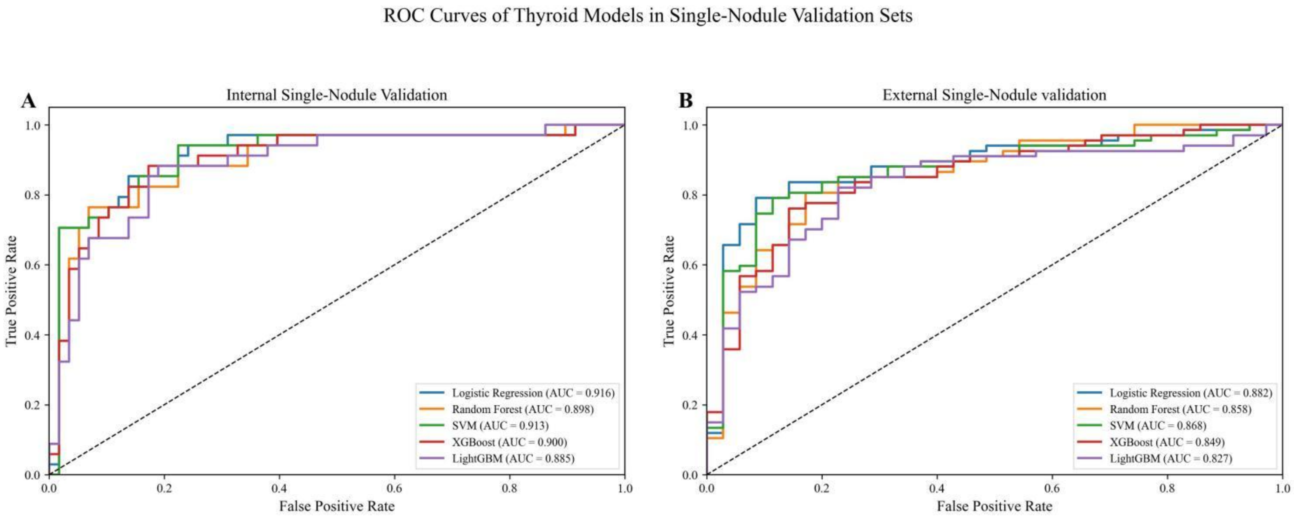

Supplementary Figure S2. (A) Internal validation cohort. (B) External validation cohort.

The ROC curves show the discrimination performance of the prediction models in patients with a single thyroid nodule. AUC values are indicated in the legend, and the dashed diagonal line represents random classification.

Supplementary Figure S3. Representative ultrasound images illustrating typical features used in C-TIRADS classification.

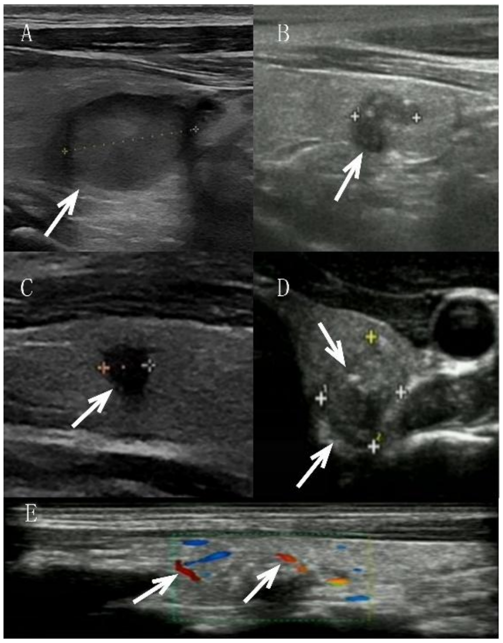

Supplementary Figure S3. (A) A benign C-TIRADS 3 nodule showing isoechogenicity and smooth margins. (B) A malignant nodule initially classified as C-TIRADS 3, demonstrating overlapping and non-specific ultrasound features that may be difficult to distinguish from benign nodules. (C) A C-

TIRADS 4a nodule with mildly suspicious features. (D) A C-TIRADS 4c nodule showing marked hypoechogenicity, irregular margins, and microcalcifications. (E) Color Doppler ultrasound image demonstrating intranodular vascularity as an example of auxiliary ultrasound information.

Supplementary Figure S4. Spearman correlation heatmap of predictors included in the final multivariable model.

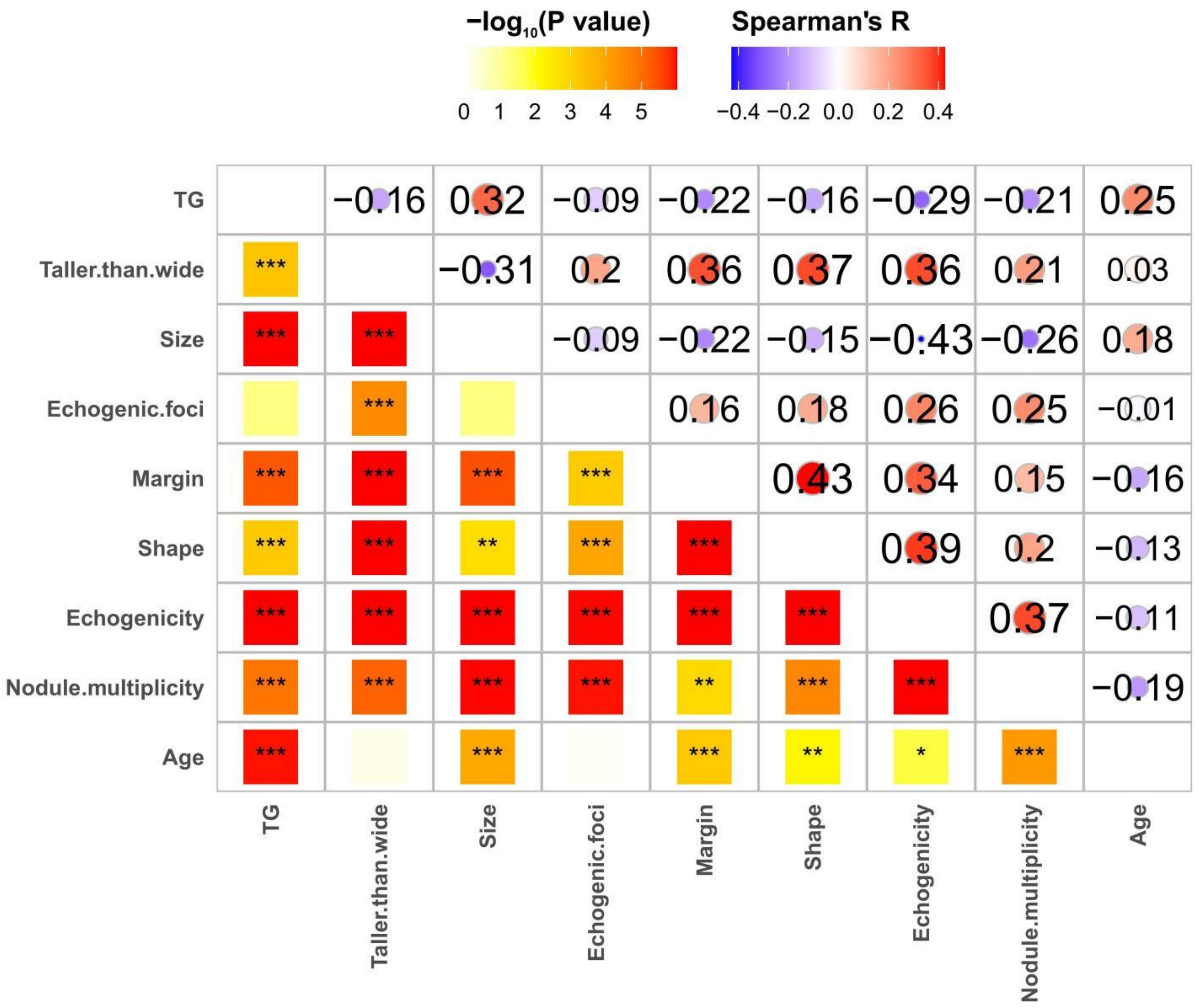

Supplementary Figure S4. Spearman correlation coefficients (R) among predictors retained in the final model were calculated in the training cohort. Color intensity represents the strength and direction of correlations (red, positive; blue, negative). Statistical significance is indicated by −log10(P value) and asterisks (*P < 0.05, **P < 0.01, ***P < 0.001).

1.2 Supplementary Tables

Supplementary Table S1. Variable retention across different stages of model development

Step	Method	Variables retained
Initial	Candidate variables (n = 20)	Age; Sex; Nodule multiplicity; Echogenicity; Shape; Margin; Echogenic foci; Size; taller-than-wide; Calcification; Blood flow; PTH; TSH; FT3; FT4; TT3; TT4; TG; TGAb; TPOAb
Descriptive analysis	Univariable analysis (descriptive only)	All candidate variables
Feature selection	LASSO regression (10-fold CV, λ1se)	Age; Nodule multiplicity; Echogenicity; Shape; Margin; Echogenic foci; Size; taller-than-wide; TG
Final	Multivariable logistic regression model (final predictors, n = 9)	Age; Nodule multiplicity; Echogenicity; Shape; Margin; Echogenic foci; Size; taller-than-wide; TG

Supplementary Table S1. LASSO, least absolute shrinkage and selection operator; CV, cross-validation. All predefined candidate variables (n = 20), including clinical characteristics, ultrasonographic features, and laboratory indicators, were simultaneously entered into the LASSO logistic regression model for feature selection. The penalty parameter (λ) was determined using 10-fold cross-validation, and the λ 1se criterion was adopted to obtain a more parsimonious and stable model. Variables with non-zero coefficients at λ 1se were retained for the final multivariable logistic regression model.

Supplementary Table S2. Hyperparameter search space and selected optimal parameters for each model.

Model	Hyperparameter	Search Range	Optimal Value
**Logistic Regression**	C	0.01, 0.1, 1, 10, 100	0.01
	penalty	L1, L2	L2
	solver	liblinear, lbfgs	liblinear
**Random Forest**	n_estimators	100, 200, 500	500
	max_depth	None, 3, 5, 10	5
	min_samples_split	2, 5, 10	2
	min_samples_leaf	1, 2, 4	1

**SVM (RBF kernel)**	C	0.1, 1, 10, 100	1
	gamma	scale, auto, 0.001, 0.01, 0.1	0.01
	kernel	rbf	rbf
**XGBoost**	n_estimators	100, 200, 500	200
	learning_rate	0.01, 0.05, 0.1	0.01
	max_depth	3, 5, 7	5
	subsample	0.7, 0.8, 1.0	0.7
	colsample_bytree	0.7, 0.8, 1.0	0.7
**LightGBM**	n_estimators	100, 200, 500	200
	learning_rate	0.01, 0.05, 0.1	0.01
	max_depth	-1, 5, 7	5
	num_leaves	15, 31, 63	15
	subsample	0.7, 0.8, 1.0	0.7

Supplementary Table S2. Hyperparameter search ranges and the final selected optimal parameters for each machine learning model. Hyperparameter tuning was performed within the training cohort using grid search combined with 10-fold cross-validation. The optimal parameter combination was selected based on the highest mean AUC across cross-validation folds.

Supplementary Table S3. Distribution of malignancy according to C-TIRADS category

C-TIRADS category	Total nodules	Benign	Malignant	Malignancy rate (%)
3	317	290	27	8.5
4a	181	76	105	58.0
4b	215	30	185	86.0
4c	111	6	105	94.6

**Total**	**824**	**402**	**422**	**51.2**

Supplementary Table S3. Malignancy rate was calculated as the proportion of malignant nodules within each C-TIRADS category. Final pathological diagnosis after surgery served as the reference standard for nodule classification. C-TIRADS: Chinese Thyroid Imaging Reporting and Data System.

Supplementary Table S4. Baseline characteristics of the training, internal validation, and external validation cohorts

Variable		Training cohort	Internal validation cohort	External validation cohort	P value*
Age		51.67 ± 12.22	54.31 ± 10.41	47.72 ± 11.90	< 0.001
Sex	Female	280	99	154	0.196
	Male	65	34	39	
Nodule multiplicity	Multiple	127	73	65	0.052
	Solitary	312	116	128	
Echogenicity	Iso-/hyperechoic	136	69	62	0.368
	Hypoechoic	306	120	131	
Shape	Oval or round	321	166	79	＜0.001
	Irregular	121	23	114	
Margin	Smooth or well-defined	338	155	86	＜0.001
	Ill-defined, lobulated, or spiculated	104	34	107	
Echogenic foci	Absent	328	142	176	＜0.001

Variable		Training cohort	Internal validation cohort	External validation cohort	P value*
	Present	114	47	17	
Size		15.53 ± 13.14	15.19 ± 13.15	13.55 ± 13.10	0.212
Taller-than-wide	No	341	153	153	0.543
	Yes	101	36	40	
Calcification	Absent	373	166	177	0.045
	Present	68	23	16	
Blood flow	Absent or peripheral	272	98	115	0.075
	Increased intranodular flow	170	91	78	
PTH		52.64 ± 63.77	50.48 ± 52.08	58.98 ± 21.39	0.261
TSH		1.99 ± 2.34	1.92 ± 1.80	3.78 ± 8.52	< 0.001
FT3		5.34 ± 4.67	4.86 ± 1.21	5.18 ± 0.87	0.286
FT4		12.04 ± 6.75	12.60 ± 6.87	11.55 ± 5.46	0.282
TT3		2.02 ± 6.99	1.49 ± 0.45	1.62 ± 0.44	0.417
TT4		138.15 ± 38.27	145.18 ± 34.07	116.51 ± 33.02	< 0.001
TG		65.52 ± 124.45	69.84 ± 132.43	50.96 ± 94.94	0.254
TGAb		32.47 ± 144.54	28.14 ± 96.12	18.20 ± 51.99	0.376
TPOAb		60.76 ± 185.84	64.70 ± 190.32	37.46 ± 147.95	0.243

Supplementary Table S4. Continuous variables are summarized as mean ± standard deviation, whereas categorical variables are reported as counts. P values were generated for descriptive reference using one-way analysis of variance or the chi-square test, depending on variable type.

Supplementary Table S5. Variance inflation factor (VIF) values for predictors included in the final multivariable logistic regression model.

Variable	VIF
Age	1.114
Nodule multiplicity	1.075
Echogenicity	1.223
Shape	1.225
Margin	1.230
Echogenic foci	1.071
Size	1.171
taller-than-wide	1.208
TG	1.062

Supplementary Table S5. VIF, variance inflation factor. VIF values were calculated in the training cohort to assess potential multicollinearity among predictors included in the final multivariable logistic regression model. All variables showed low VIF values (range, 1.062 – 1.230), indicating no evidence of significant multicollinearity.

Supplementary Table S6. Variable definitions and coding

Variable	Definition	Coding/Measurement
Age	Age at diagnosis	Continuous, years
Sex	Biological sex of the patient	Female = 0; Male =1
Nodule multiplicity	Number of thyroid nodules detected by ultrasound	Multiple = 0；

Echogenicity	Echogenicity of the thyroid nodule relative to surrounding thyroid tissue	Iso-/hyperechoic = 0;
Shape	Shape of the thyroid nodule on ultrasound	Oval or round = 0; Irregular = 1
Margin	Margin characteristics of the thyroid nodule	Smooth or well-defined = 0;
Echogenic foci	Presence of echogenic foci within the thyroid nodule	Absent = 0; Present = 1
Size	Maximum diameter of the thyroid nodule	Continuous, millimeters (mm)
Taller-than-wide	Anteroposterior dimension greater than transverse dimension on ultrasound	No = 0; Yes = 1
Calcification	Presence of calcification within the thyroid nodule on ultrasound	Absent = 0; Present = 1
Blood flow	Intralesional blood flow of the thyroid nodule assessed by Doppler ultrasound	Absent or peripheral = 0; Increased intranodular flow = 1
PTH	Parathyroid hormone level	Continuous, pg/mL
TSH	Thyroid-stimulating hormone level	Continuous, mIU/L
FT3	Free triiodothyronine level	Continuous, pmol/L
FT4	Free thyroxine level	Continuous, pmol/L
TT3	Total triiodothyronine level	Continuous, nmol/L
TT4	Total thyroxine level	Continuous, nmol/L
TG	Serum thyroglobulin level	Continuous, ng/mL
TGAb	Thyroglobulin antibody level	Continuous, IU/mL
TPOAb	Thyroid peroxidase antibody level	Continuous, IU/mL

Supplementary Table S6. Supplementary Table S6 presents the definitions and coding of all candidate clinical, ultrasonographic, and laboratory variables considered for model development. Continuous variables were entered into the models without categorization, while categorical variables were coded as binary indicators according to predefined criteria. For patients with multiple nodules, patient-level variables were assigned to each nodule.

Statistical Analysis. Restricted cubic spline analyses were performed to assess potential non-linear associations between key continuous predictors and the outcome. No clinically meaningful non-linearity was observed; therefore, spline plots are not presented.
